# Complement Component C1q Programs a Pro-Efferocytic Phenotype while Limiting TNFα Production in Primary Mouse and Human Macrophages

**DOI:** 10.3389/fimmu.2016.00230

**Published:** 2016-06-15

**Authors:** Holly J. Hulsebus, Sean D. O’Conner, Emily M. Smith, Chunfa Jie, Suzanne S. Bohlson

**Affiliations:** ^1^Department of Microbiology and Immunology, Des Moines University, Des Moines, IA, USA; ^2^Office of Research, Des Moines University, Des Moines, IA, USA

**Keywords:** complement, C1q, inflammation, macrophages, lupus erythematosus, systemic, phagocytosis

## Abstract

Deficiency in complement component C1q is associated with an inability to clear apoptotic cells (efferocytosis) and aberrant inflammation in lupus, and identification of the pathways involved in these processes should reveal important regulatory mechanisms in lupus and other autoimmune or inflammatory diseases. In this study, C1q-dependent regulation of TNFα/IL-6 expression and efferocytosis was investigated using primary mouse bone marrow-derived macrophages and human monocyte-derived macrophages. C1q downregulated LPS-dependent TNFα production in mouse and human macrophages. While prolonged stimulation with C1q (18 h) was required to elicit a dampening of TNFα production from mouse macrophages, the human macrophages responded to C1q with immediate downregulation of TNFα. IL-6 production was unchanged in mouse and upregulated by human macrophages following prolonged stimulation with C1q. Our previous studies indicated that C1q programmed enhanced efferocytosis in mouse macrophages by enhancing expression of Mer tyrosine kinase and its ligand Gas6, a receptor–ligand pair that also inhibits proinflammatory signaling. Here, we demonstrated that C1q-dependent programming of human macrophage efferocytosis required protein synthesis; however, neither Mer nor the related receptor Axl was upregulated in human cells. In addition, while the C1q-collagen-like tails are sufficient for promoting C1q-dependent phagocytosis of antibody-coated targets, the C1q-tails failed to program enhanced efferocytosis or dampen TNFα production. These data further elucidate the mechanisms by which C1q regulates proinflammatory signaling and efferocytosis in macrophages, functions that are likely to influence the progression of autoimmunity and chronic inflammation.

## Introduction

C1q, well known for its function as the recognition component of the classical complement pathway, also functions independently of the complement system to facilitate phagocytosis and regulate proinflammatory signaling in myeloid cells [reviewed in Bohlson et al. (1)]. To this end, *in vitro* studies demonstrate that C1q and other members of the defense collagen family, such as mannose-binding lectin (MBL) and surfactant protein-A (SP-A), are bridging molecules that bind to target particles, such as pathogens or apoptotic cells, and facilitate their rapid clearance [reviewed in Galvan et al. (2)]. C1q deficiency is associated with the development of autoimmunity, which is thought to result from an inefficient clearance of apoptotic cells or “efferocytosis,” and apoptotic/dying cells are considered to be a source of lupus autoantigens (3). While the function of C1q as a bridging molecule that physically links the apoptotic cell to the phagocyte has been appreciated for years, more recent studies indicate an additional function for C1q in macrophage polarization. C1q programs macrophages to an anti-inflammatory and pro-efferocytic phenotype, consistent with an M2-like phenotype (4–6). Physical bridging and programming may occur concomitantly; however, little is known about the molecular mechanisms leading to C1q-dependent regulation of the macrophage phenotype.

Korb and Ahearn (7) were the first to demonstrate that C1q binds directly and specifically to apoptotic cells using human apoptotic keratinocytes. This work led to a series of supporting studies that demonstrated that C1q bridges apoptotic cells to phagocytes to facilitate the rapid removal of cellular debris in both mouse and human phagocytes [reviewed in Galvan et al. (2)]. Using primary human monocytes, macrophages and dendritic cells, Fraser and colleagues demonstrated that immobilized C1q bound to apoptotic cells inhibited LPS-dependent TNFα production in human macrophages but did not stimulate efferocytosis unless serum was present, resulting in activation of the classical complement pathway and deposition of C3b (8). In addition, C1q-opsonized autologous human apoptotic lymphocytes inhibited activation of the NLRP3 inflammasome and skewed human macrophages toward an anti-inflammatory phenotype (4). Furthermore, Clarke et al. (9) demonstrated that C1q-bound apoptotic cells suppressed human macrophage and dendritic cell-mediated TH17 and TH1 T cell subset proliferation suggesting that T-cell-mediated immune responses are altered when C1q serves as an opsonin for a target particle. Apoptotic cells alone are anti-inflammatory, and these studies suggest that C1q-opsonization further enhances the anti-inflammatory properties of apoptotic cells.

In our previous studies, to determine if C1q could program macrophages toward an anti-inflammatory and pro-efferocytic phenotype in the absence of apoptotic cells, we stimulated mouse macrophages with C1q and investigated changes in gene expression by microarray. We demonstrated that C1q stimulation resulted in expression of pro-efferocytic molecules, including Mer tyrosine kinase (Mer) and its ligand Gas6 (5). This finding is consistent with the report that expression of pro-efferocytic molecules is decreased in macrophages from human lupus patients (10). Mer is a member of the Tyro, Axl, and Mer (TAM) family of receptor tyrosine kinases that regulate efferocytosis and downregulate proinflammatory signaling in myeloid cells [reviewed in Lemke and Rothlin (11)]. C1q-dependent efferocytosis was dependent on Mer/Gas6 in mouse macrophages, and this was the first demonstration that C1q could program efferocytosis independent of its role as an opsonin (2). In the current study, we sought to further define the role of C1q in programming the macrophage phenotype. To do this, we incubated human and mouse primary macrophages on immobilized C1q prior to the addition of apoptotic cells to differentiate the role of C1q in macrophage programming from the opsonic activity of C1q and compare responses in primary mouse and human cells. The data suggest that C1q programs human and mouse macrophages toward a pro-efferocytic phenotype and also programs altered cytokine signaling. This programming may be important in autoimmune diseases, such as lupus, where dysregulated macrophage activation may contribute to chronic inflammation.

## Materials and Methods

Dulbecco’s Modified Eagle’s Medium (DMEM) and RPMI 1640 were purchased from Gibco/Life Technologies (Grand Island, NY, USA). Chemically defined, serum-free medium (HL1) was obtained from Lonza (Walkersville, MD, USA) and supplemented with 1% l-alanyl-l-glutamine (Corning, Inc., Corning, NY, USA), 10-mM HEPES and 5-mM MgCl_2_. Penicillin/streptomycin solution was purchased from Gibco/Life Technologies. Fetal bovine serum (FBS) was purchased from HyClone Laboratories/GE Healthcare Life Sciences (Logan, UT, USA) and heat inactivated at 56°C for 30 min. Bovine serum albumin (BSA) was purchased from Sigma-Aldrich (St. Louis, MO, USA). C1q was purified from normal human plasma as previously described in detail (12, 13). Briefly, pooled normal human serum was separated by ion-exchange chromatography (BioRex 70). After extensive washing, the bound C1q was eluted with a salt gradient of 0.082–0.3M NaCl containing 0.05M phosphate and 2-mM EDTA. Fractions containing C1q as determined by hemolytic assay were pooled and precipitated with 33% ammonium sulfate. The purified C1q was redissolved and separated by size-exclusion chromatography (Biogel A5m). The C1q-containing fractions were again concentrated with ammonium sulfate, dialyzed, and stored at −80°C. The purified C1q is hemolytically active, and homogeneous as assessed by SDS-PAGE. Evidence for the immunochemical purity of the C1q has been extensively described (12). The C1q preparation was free of endotoxin as determined by the LAL Chromogenic Endotoxin Quantitation Kit (Fisher Scientific, Rockford, IL, USA). Dr. Andrea Tenner (University of California, Irvine, Irvine, CA, USA) kindly provided the C1q collagen-like tails used in our studies. Human serum albumin (HSA) was purchased from Baxter (Deerfield, IL, USA). Cycloheximide was purchased from Chem Service (West Chester, PA, USA) and dissolved in endotoxin-free water at a concentration of 25 mM. CFSE was purchased from Invitrogen/Life Technologies (Grand Island, NY, USA) and reconstituted to 5 mM in DMSO. Etoposide (Sigma) was reconstituted to 10 mM in DMSO. Ultra-pure LPS from *Escherichia coli* 055.B5 was from List Biological Laboratories (Campbell, CA, USA).

### Antibodies

Rabbit anti-mouse Mer primary antibody and goat anti-mouse Axl were purchased from Santa Cruz Biotechnology (Santa Cruz, CA, USA). Rabbit anti-human Mer primary antibody was purchased from Abcam (Cambridge, MA, USA) and goat anti-human Axl was purchased from R&D Systems (Minneapolis, MN, USA). Mouse anti-actin primary antibody was purchased from Sigma-Aldrich. HRP-conjugated secondary antibodies were purchased from Jackson ImmunoResearch (West Grove, PA, USA). Isotype control and phycoerythrin-tagged CD11b antibodies were purchased from eBioscience.

### Mice

All mice were of a C57Bl/6 background. Mice were housed in a pathogen-free facility in the Des Moines University animal care facility. All methods were performed in accordance with protocols approved by the Des Moines University Institutional Animal Care and Use Committee.

### Mouse Cell Culture

Bone marrow-derived macrophages (BMDM) were generated as previously described (14). Femurs and tibias were removed from 6- to 12-week-old C57Bl/6, and bone marrow was flushed from the bones with DMEM containing 5% heat-inactivated FBS, 10-mM HEPES, and 100 units/ml penicillin G sodium/100 μg/ml streptomycin sulfate (Pen/Strep). Cells were cultured in DMEM containing 5% heat-inactivated FBS, 10-mM HEPES, and Pen/Strep at 37°C and 5% CO_2_ for 2 h to remove any resident fibroblasts or macrophages. Non-adherent cells were cultured in BMDM medium (DMEM, 15% L929 conditioned medium, 10% heat-inactivated FBS, Pen/Strep, and 10-mM HEPES) at 37°C and 5% CO_2_ for 4 days, and media was replaced. Cells were fully mature by day 7. Media was replaced every 2–3 days and cells were passed every 7 days to maintain viability (15).

### Human Cell Culture

Heparinized whole blood (100 ml) was obtained by venipuncture from healthy human donors, diluted 1:1 in endotoxin-free sterile saline, and separated into components by gradient centrifugation using lymphocyte separation medium (MP Biomedicals, Solon, OH, USA). Mononuclear cells were collected, washed three times with RPMI 1640, and the monocytes were isolated through negative selection using magnetic Dynabeads according to the manufacturer’s protocol (Invitrogen/Life Technologies). Monocytes were cultured in RPMI supplemented with 20% human AB+ serum (Fisher Scientific) and Pen/Strep at 37°C and 5% CO_2_. After a 4-day maturation period, media was replaced and human monocyte-derived macrophages (HMDM) were harvested for assays on day 7. The human Jurkat T cell line was purchased from ATCC (Manassas, VA, USA) and cultured in RPMI supplemented with 10% heat-inactivated FBS and Pen/Strep. All procedures were approved by the Des Moines University Institutional Review Board before initiation, and all procedures performed were in accordance with the approved protocol.

### Phagocytosis Assays

Sheep erythrocytes were suboptimally opsonized with rabbit anti-sheep IgG (EAIgG) as previously described (16). Briefly, anti-sheep IgG was isolated from anti-sheep hemolysin (Colorado Serum Company, Denver, CO, USA) using GammaBind G Sepharose (GE Healthcare, Pittsburgh, PA, USA). EAIgG phagocytosis assays were performed as described (17). Percent phagocytosis was calculated as the number of macrophages that ingested at least one EAIgG divided by the total number of macrophages counted, multiplied by 100. At least 200 macrophages per well were scored by microscopy under oil immersion and by individuals blinded to the experimental conditions; all experimental conditions were performed in duplicate.

For clarity throughout the manuscript, assays that measured engulfment of EAIgG are referred to as “phagocytosis,” whereas assays that measured the engulfment of apoptotic cells are referred to as “efferocytosis.” Efferocytosis assays were performed as follows: four-well Lab-Tek Chamber Slides (Nunc/Thermo Fisher Scientific, Rochester, NY, USA) were coated with 400 μl of full-length C1q, C1q collagen-like tails, or human serum albumin (HSA) at 4–8 μg/ml for 2 h at room temperature. Concentrations of 4 and 8 μg/ml stimulate C1q-dependent functions without significant changes in background (HSA) activity. The wells were gently washed twice with PBS before adding 500 μl of macrophages at a concentration of 5 × 10^5^/ml in supplemented HL1. Cells were allowed to adhere for the times indicated in figure legends. Jurkat cells were labeled with 5 μM CFSE for 30 min at 37°C and washed with complete media twice before the addition of 40 μM etoposide for 15 h to induce apoptosis. Apoptotic Jurkats were washed twice with phagocytosis buffer (RPMI supplemented with 5-mM MgCl_2_ and Pen/Strep) before being added at a 3:1 ratio of targets to macrophages. Slides were centrifuged at 700 rpm for 3 min and incubated at 37°C and 5% CO_2_ for 1 h. After incubation, slides were gently washed with PBS twice to remove non-ingested targets, and cells were trypsinized, collected into FACS buffer (Hank’s Balanced Salt Solution supplemented with 0.2% BSA), washed, and stained with CD11b-PE antibody. Efferocytosis assays were analyzed by flow cytometry as described (17), and percent efferocytosis was calculated as the number of cells positive for both CD11b-PE and CFSE, divided by the total number of CD11b-PE-positive cells, multiplied by 100. Percentage of apoptotic cells was measured for each assay using Annexin V and propidium iodide staining (Table S1 in Supplementary Material).

### Western Blotting

Lab-Tek chamber slides were coated with 2 ml of 4–8 μg/ml HSA or C1q for 2 h before the addition of 10^6^ BMDM or 5 × 10^5^ HMDM in supplemented HL1. At the indicated time points, whole-cell lysates were collected with RIPA buffer (50 mM Tris–HCl pH 7.4, 150 mM NaCl, 0.5% sodium deoxycholate, 0.1% SDS, and 1% Triton-X) supplemented with 10 mM sodium fluoride, 2 mM EDTA, 1 mM PMSF, and protease and phosphatase inhibitor tablets (Roche, Indianapolis, IN, USA). Total protein concentration was calculated by BCA assay according to the manufacturer’s protocol (Thermo Scientific, Rockford, IL, USA), and 14–20 μg protein was resolved using 10% SDS-PAGE under reducing conditions. Protein samples were transferred to a PVDF membrane and blocked for at least 1 h. Membranes were then probed with antibodies as described in the figure legends and developed using enhanced chemiluminescent reagents (GE Healthcare).

### Cytokine Assays

Lab-Tek chamber slides were coated with HSA, C1q, or C1q collagen-like tails at 4–8 μg/ml for 2 h. BMDM or HMDM were harvested using PBS containing 10 mM EDTA, washed, and resuspended in supplemented HL1. The 5 × 10^5^ HMDM or 10^6^ BMDM were added to the wells and allowed to adhere for 30 min or 18 h. Cells were then activated with LPS (300 ng/ml) for 4 h. Supernatants were collected, and TNFα and/or IL-6 were quantified by Ready-Set-Go ELISA kits, according to the manufacturer’s protocol (eBioscience).

### Statistical Analysis

**Figure 1:**
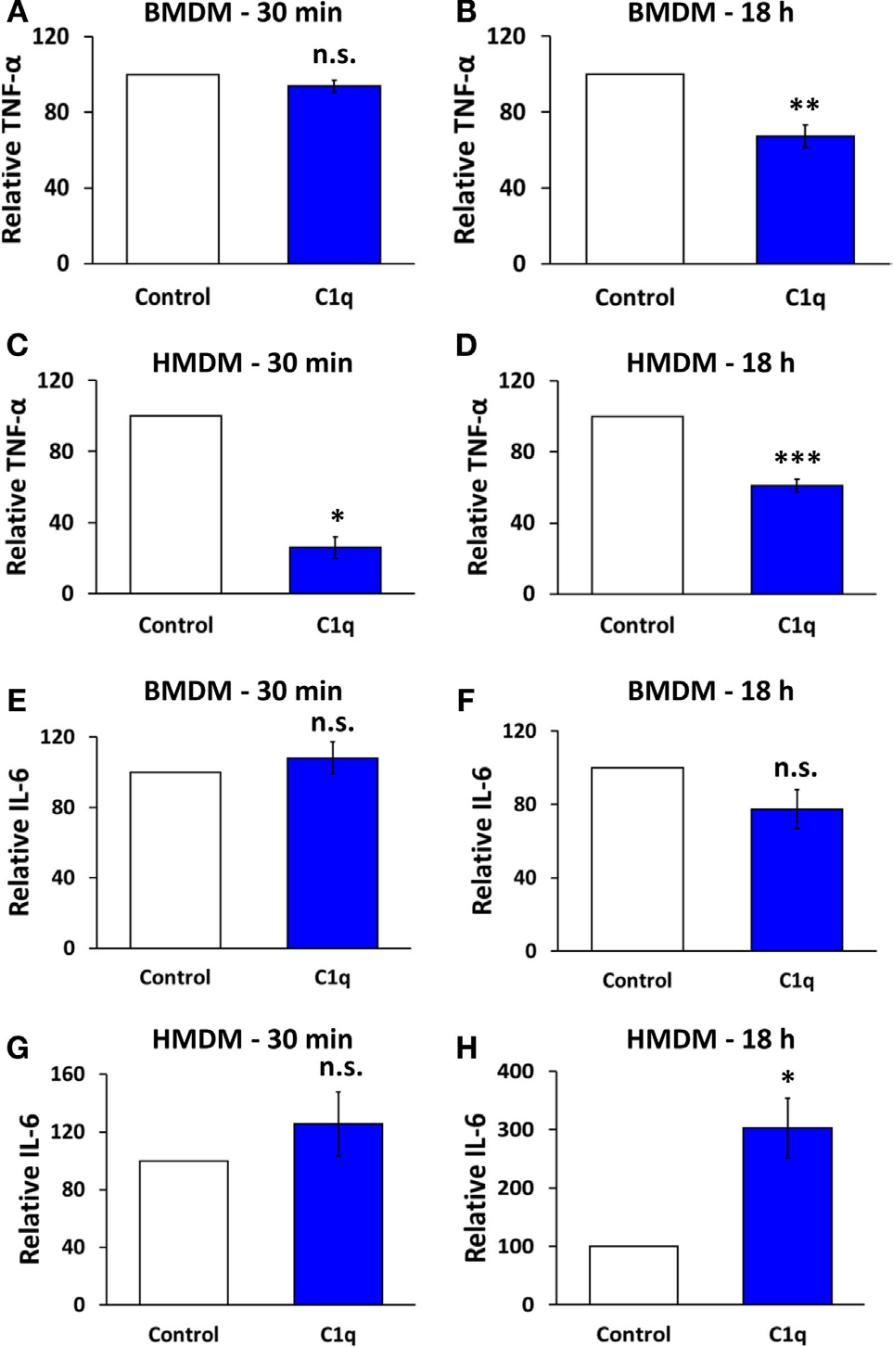
**C1q programs an alteration in cytokine signaling in mouse bone marrow-derived macrophages and human monocyte-derived macrophages**. BMDM or HMDM were adhered to C1q- or HSA (control)-coated Lab-Tek Chamber (LTC) slides for 30 min or 18 h, as indicated in individual graph titles. Cells were then stimulated with 300 ng/ml LPS for 4 h and TNF-α or IL-6 was quantified by ELISA from supernatants according to manufacturer’s protocol. Bars represent the average cytokine production of C1q relative to HSA from three **(E,G,H)**, four **(C)**, five **(D)**, six **(A)**, seven **(F)**, or eight **(B)** individual experiments ± SEM. *p < 0.05; **p < 0.01; ***p = 0.001; n.s. = not significant.

**Figure 2:**
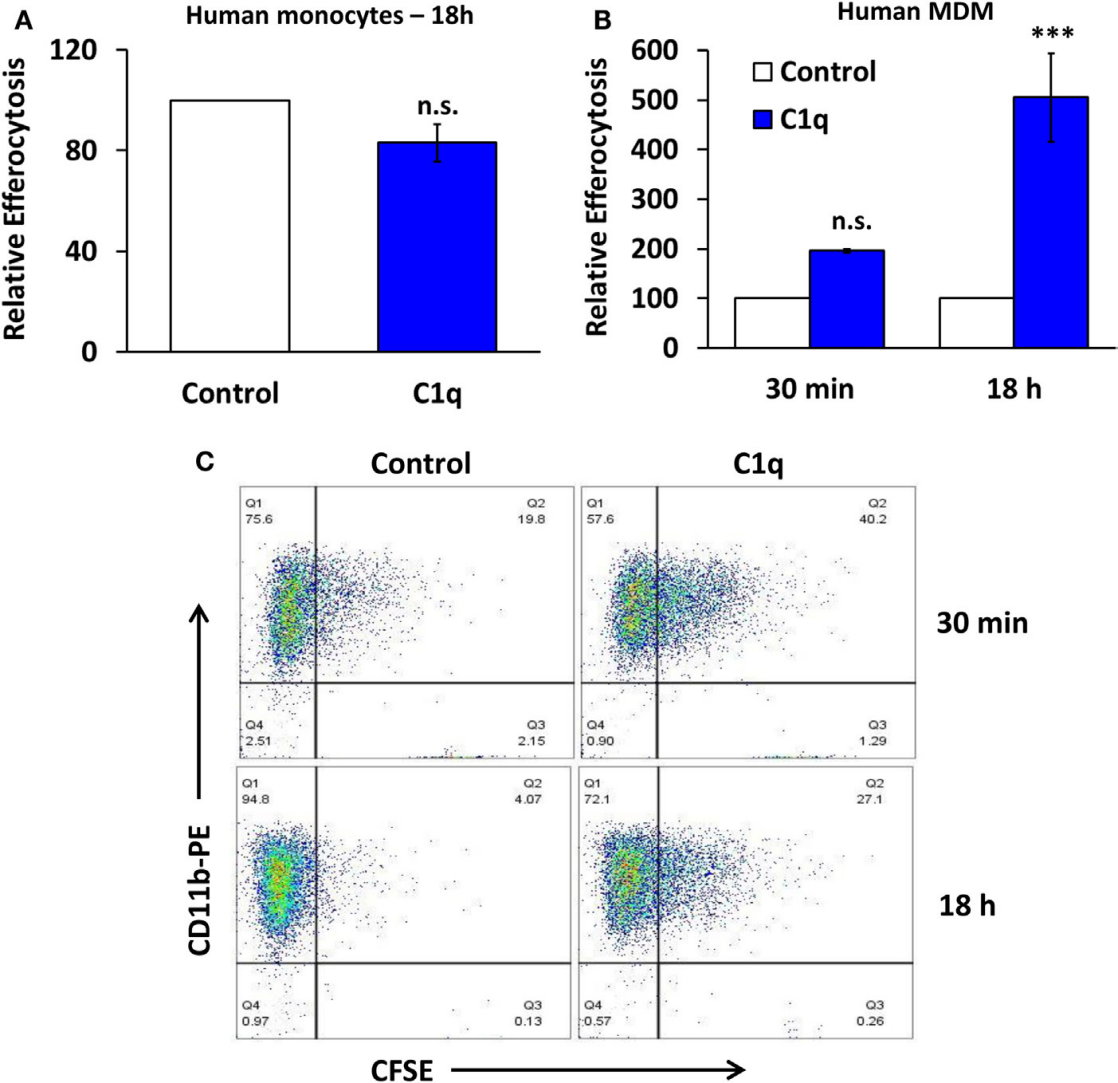
**C1q stimulates programmed efferocytosis in human monocyte-derived macrophages, but not primary human monocytes**. **(A)** Primary human monocytes were cultured in LTC slides coated with 8 μg/ml HSA (control) or C1q for 18 h and fed CFSE-labeled apoptotic Jurkats at a 3:1 ratio of targets to macrophages for 1 h. Efferocytosis was quantified by flow cytometry. Bars represent average efferocytosis of C1q relative to HSA from three experiments ± SEM. n.s. = not significant. **(B)** Human monocyte-derived macrophages (MDM) were cultured as in **(A)** for 30 min or 18 h and fed CFSE-labeled apoptotic Jurkats at a 3:1 ratio of targets to macrophages for 1 h. Efferocytosis was quantified by flow cytometry. Bars represent average efferocytosis of C1q relative to HSA from three experiments (30 min) or four experiments (18 h) ± SEM. n.s. = not significant (*p* = 0.052); ***p < 0.001. **(C)** Representative flow cytometry scatter plots from one experiment as described in **(B)**.

Due to variability in total cytokine levels between human donors and mice, descriptive statistics in Figures 1, 2, 4 and 5 were presented as relative means from combined experiments with SEs. For phagocytosis and efferocytosis experiments that were collected as counts/percentage data, an arcsine square root transformation was first performed. For better approximation to normal distribution, the Western blot data in Figure 4B were normalized to the internal controls as ratios and further log transformed on the base 2 log scale before the linear model analysis below. The cytokine (ELISA) data in Figures 1 and 5 were log transformed on the base 2 log scale. Analysis of variance (ANOVA) was then performed on the properly transformed data sets using a linear model that accounts for the different sources of variation, treatment, and experiment. The comparisons of interest were made using statistical contrasts of the treatment factor in the ANOVA. Multiple comparison adjustment was made with Bonferroni or Tukey procedure where appropriate. The *p*-value < 0.05 is considered to be significant.

## Results

### C1q Programs an Alteration in Cytokine Signaling in Mouse Bone Marrow-Derived Macrophages and Human Monocyte-Derived Macrophages

Previous studies have shown that C1q provides a direct inhibition of proinflammatory signaling in a variety of myeloid cells (4, 6, 8, 18–21). However, when mouse BMDM were stimulated with C1q for 30 min and then activated with lipopolysaccharide (LPS), control, and C1q-stimulated BMDM secreted equivalent levels of TNFα (Figure 1A). In contrast, BMDM that were stimulated with C1q for 18 h secreted significantly less TNFα compared to control cells (Figure 1B, range of inhibition 12.4–55.9%, *n* = 8). This suggests that BMDM require a C1q-dependent programming event in order to respond to C1q with this diminished proinflammatory response. Similar experiments were performed with HMDM, and unlike BMDM, there was a direct inhibition of proinflammatory signaling in the HMDM; HMDM stimulated with C1q for 30 min and then activated with LPS produced significantly less TNFα compared to control cells (Figure 1C, range of inhibition 60.2–87.6%, *n* = 4). In addition, HMDM that were stimulated with C1q for 18 h prior to treatment with LPS produced significantly less TNFα compared to control cells (Figure 1D, range of inhibition 30.9–40.4%, *n* = 5). Importantly, both BMDM and HMDM that were not stimulated with LPS produced little or no proinflammatory cytokines (Figure S1 in Supplementary Material). While IL-6 production was not significantly altered in BMDM after 30 min or 18 h stimulation with C1q (Figures 1E,F), IL-6 production was enhanced in HMDM. C1q-stimulated HMDM produced equivalent amounts of IL-6 compared to control after 30 min stimulation (Figure 1G), but produced a significantly higher amount of IL-6 following 18 h programming with C1q. There was a 2.2- to 3.9-fold increase in IL-6 production in HMDM pre-stimulated with C1q for 18 h (Figure 1H, *n* = 3). These data demonstrate that C1q regulates IL-6 and TNFα production in macrophages, and some activities require prolonged programming with C1q.

### C1q Stimulates Programmed Efferocytosis in Mouse and Human Macrophages and Is Dependent on Protein Synthesis

**Figure 3:**
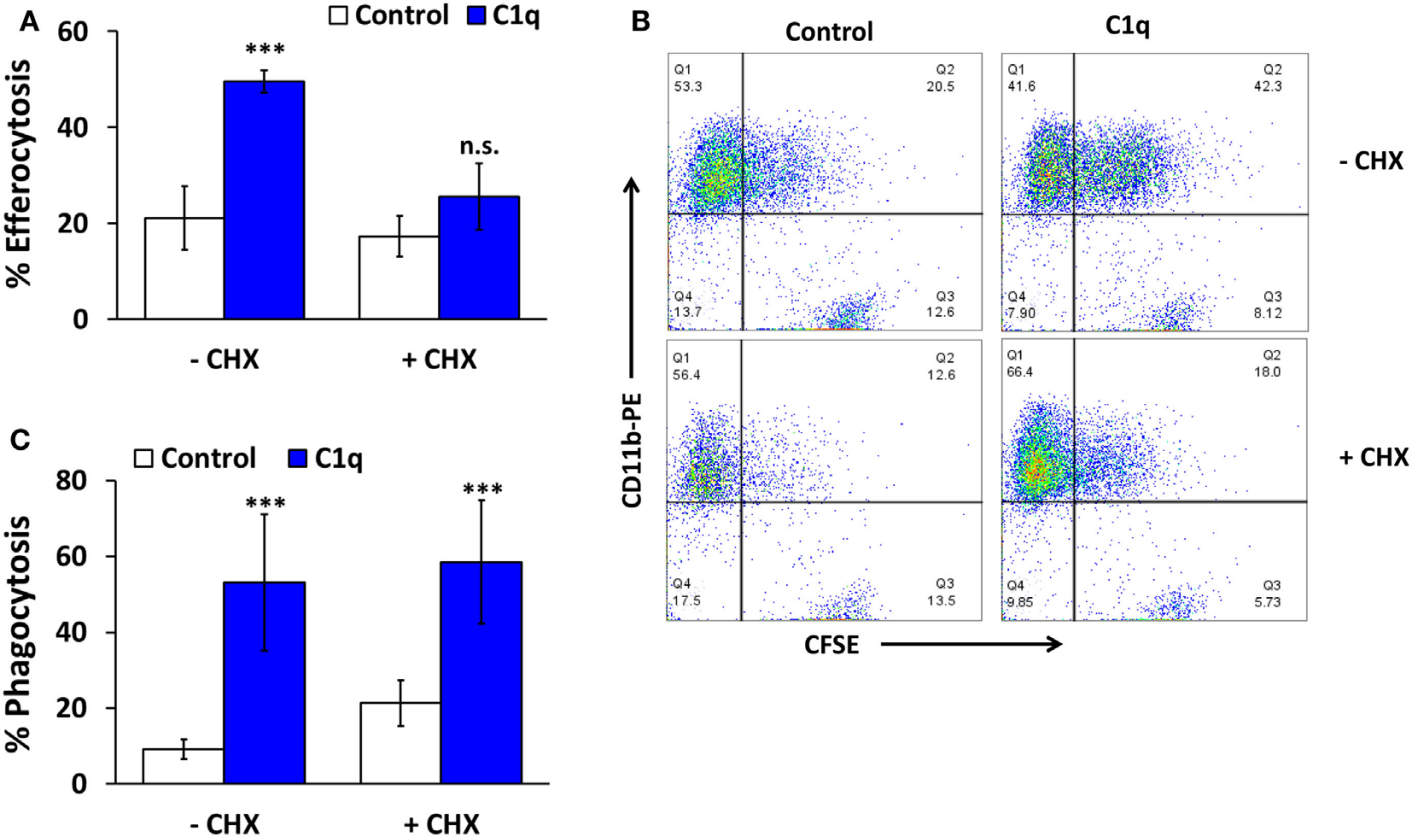
**C1q-dependent efferocytosis requires protein synthesis**. **(A)** HMDM were adhered to LTC slides coated with 8 μg/ml HSA (control) or C1q for 30 min and 100 μM cycloheximide (CHX) was added for 4 h. CFSE-labeled apoptotic Jurkats were then added at a 3:1 ratio of targets to macrophages for 1 h and efferocytosis was quantified by flow cytometry. Bars represent the mean of four individual experiments ± SEM. ***p < 0.001; n.s. = not significant. **(B)** Representative flow cytometry scatter plots from an experiment as described in **(A)**. **(C)** HMDM were cultured in LTC slides coated with 8 μg/ml HSA (control) or C1q for 30 min and fed EAIgG for 30 min. Cells were fixed and at least 200 cells/well from duplicate wells were scored by microscopy. Bars represent the mean of three individual experiments ± SEM. ***p < 0.001.

Our previous studies demonstrated that C1q-programmed mouse BMDM to express pro-efferocytic molecules, and as a result there was enhanced efferocytic activity in C1q-programmed BMDM. The enhanced efferocytic activity required C1q-dependent expression of Mer tyrosine kinase (Mer) and its ligand Gas6 (2). To determine if C1q-dependent programmed efferocytosis occurred in human cells, human monocytes and HMDM were stimulated with C1q for 18 h, and efferocytosis was measured. C1q-programmed monocytes engulfed the same number of apoptotic cells as the control monocytes (Figure 2A, *n* = 3). In contrast, HMDM engulfed twofold more apoptotic cells compared to control cells after a 30-min stimulation with C1q and greater than fivefold after 18 h of C1q stimulation (Figure 2B, *n* = 3 and Figure 2C, *n* = 4). These data suggested that the maturation state and culture conditions of the macrophage dictated its ability to respond to C1q with programmed efferocytosis. Since programmed efferocytosis in BMDM requires expression of Mer and Gas6, we predicted that *de novo* protein synthesis was required for programmed efferocytosis in HMDM. To test the requirement for protein synthesis, HMDM were treated with and without the protein synthesis inhibitor cycloheximide prior to being fed apoptotic targets. Cycloheximide inhibited C1q-dependent enhanced efferocytosis in HMDM demonstrating that protein synthesis is required for programmed efferocytosis in HMDM (Figures 3A,B, *n* = 4). C1q triggers a direct enhancement of phagocytosis of antibody-coated particles which occurs within 5 min of stimulation with C1q (18). As expected, cycloheximide treatment did not inhibit C1q-dependent enhanced engulfment of antibody-coated targets indicating that the cycloheximide did not inhibit all C1q-dependent pathways, or phagocytosis in general (Figure 3C, *n* = 3).

### Mer and Axl Are Upregulated in Mouse Macrophages but Not Human Macrophages

**Figure 4:**
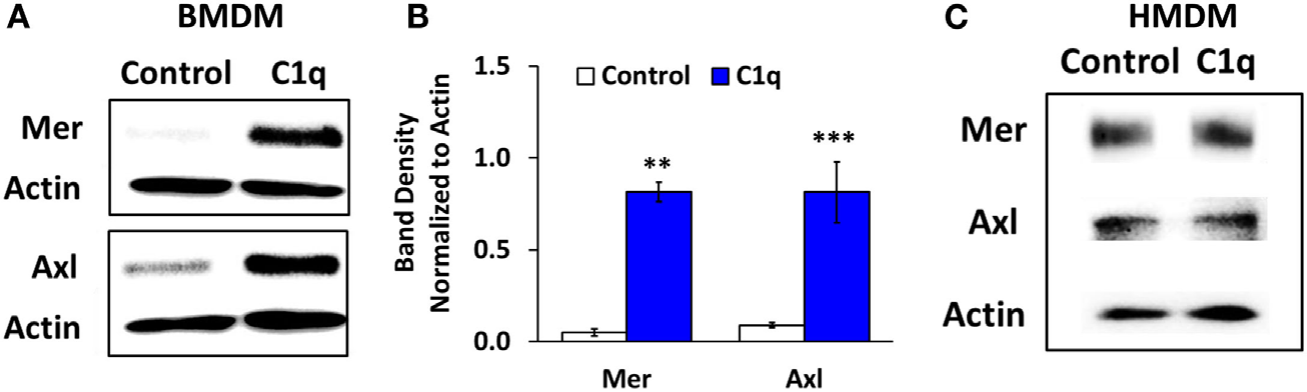
**Mer and Axl expression are upregulated after prolonged C1q stimulation in BMDM, but not HMDM**. **(A)** BMDM were adhered to LTC slides coated with 4 μg/ml HSA (control) or C1q for 18 h and Mer and Axl were detected from lysates by western blot. Actin was measured as a loading control. Blot is representative of three separate experiments. **(B)** Densitometric analysis of experiments (ImageJ) described in **(A)**, normalized to actin ± SEM. **p < 0.01; ***p = 0.001 **(C)** HMDM were cultured in LTC slides coated with 8 μg/ml HSA (control) or C1q for 18 h and Mer and Axl expression were detected from lysates by western blot. Actin was measured as a loading control. Blot is representative of two separate experiments.

Since Mer is required for programmed efferocytosis in BMDM, and Mer and other members of the TAM family of receptor tyrosine kinases also downregulate proinflammatory signaling, we evaluated the expression of Mer in C1q-dependent regulation of proinflammatory signaling. As shown previously in Ref. (5) and represented in Figures 4A,B, 18-h stimulation with C1q resulted in upregulated expression of Mer in BMDM. The related TAM family kinase, Axl, was also upregulated in C1q-stimulated BMDM (Figures 4A,B, *n* = 3). However, the HMDM failed to upregulate Mer and Axl when stimulated with C1q for 18 h (Figure 4C; Figure S2 in Supplementary Material). These studies suggest that unlike BMDM, programmed efferocytosis in HMDM is independent of Mer (or Axl) upregulation.

### Programmed Efferocytosis and Inhibition of Proinflammatory Signaling Requires Full-length C1q

**Figure 5:**
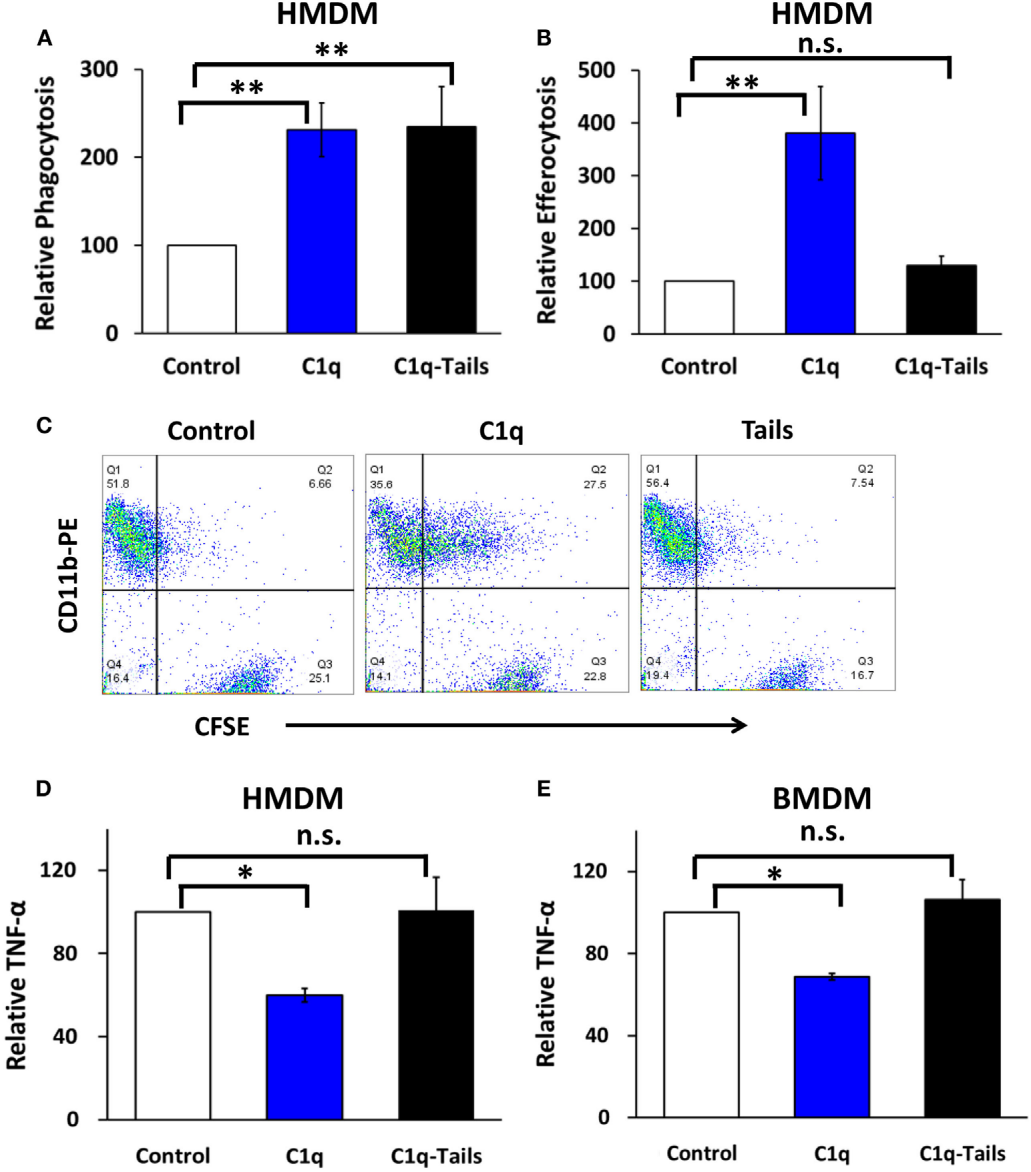
**Programmed efferocytosis and inhibition of proinflammatory signaling requires full-length C1q**. **(A)** HMDM were adhered to LTC slides coated with 8 μg/ml HSA (control), C1q, or C1q-Tails for 30 min and fed EAIgG. Phagocytosis was allowed to occur for 30 min and percent phagocytosis was measured from at least 200 cells/well from duplicate wells. Bars represent the mean of three separate experiments normalized to control ± SEM. **p < 0.01. **(B)** HMDM were cultured in LTC slides coated with 8 μg/ml HSA (control), C1q, or C1q-Tails for 18 h and fed CFSE-labeled apoptotic Jurkats at a 3:1 ratio of targets to macrophages for 1 h. Efferocytosis was quantified by flow cytometry. Bars represent the mean of three individual experiments normalized to control ± SEM. **p < 0.01; n.s. = not significant **(C)** Representative flow cytometry scatter plots from an experiment as described in **(B)**. **(D)** HMDM were adhered to LTC slides coated with 8 μg/ml HSA (control), C1q, or C1q-Tails for 18 h and stimulated with 300 ng/ml LPS for 4 h. TNF-α was quantified from supernatants by ELISA. Bars represent the mean of three separate experiments normalized to control ± SEM. *p < 0.05; n.s. = not significant **(E)** BMDM were adhered to LTC slides coated with 4 μg/ml HSA (control), C1q, or C1q-Tails for 18 h and stimulated with 300 ng/ml LPS for 4 h. TNF-α was quantified from supernatants by ELISA. Bars represent the mean of three separate experiments normalized to control ± SEM. *p < 0.05; n.s. = not significant.

C1q stimulates a direct enhancement of phagocytosis of antibody-opsonized targets, and this pathway is activated by the C1q collagen-like tails. Arora et al. (22) identified a conserved six-amino acid sequence in the collagen-like tail of C1q (and related defense collagens) that is required for the phagocytic function. In our previous studies, the collagen-like tail failed to program enhanced efferocytosis in BMDM (2). To determine if the collagen-like tails were sufficient for the regulation of proinflammatory signaling, we confirmed that the tails were functional in a phagocytosis assay using antibody-opsonized sheep red blood cells (EAIgG). C1q-tails and full-length C1q stimulated comparable levels of phagocytosis of EAIgG in HMDM (Figure 5A). C1q-tails stimulated 1.4- to 3-fold enhanced phagocytosis of EAIgG, and C1q stimulated 1.7- to 2.6-fold enhanced phagocytosis of EAIgG (*n* = 3). Similar to BMDM, programmed efferocytosis in HMDM required the full-length molecule since the collagen-like tails failed to stimulate enhanced efferocytosis (Figure 5B). C1q treatment resulted in a 3.8-fold enhancement of efferocytosis in HMDM, whereas there was not a significant difference in engulfment between control cells and cells treated with the C1q-tails (*n* = 3). In addition, while full-length C1q programmed a downregulation of LPS-dependent TNFα production, the collagen-like tails failed to downregulate proinflammatory signaling in both human (Figure 5D) and mouse (Figure 5E) macrophages. There was a 34–45% range of inhibition of TNFα production from human macrophages treated with C1q (*n* = 3) and a 28–33% range of inhibition of TNFα production from mouse macrophages (*n* = 3). No significant difference in TNFα production was detected between human or mouse control macrophages and C1q-tails-treated macrophages. These data indicate that the C1q collagen-like tails are insufficient for mediating C1q-dependent programming of enhanced efferocytosis and inhibition of TNFα production.

## Discussion

The objective of this study was to investigate the role of programming on C1q-dependent enhancement of efferocytosis and inhibition of proinflammatory signaling in mouse and human primary macrophages. To accomplish this objective, we used mouse BMDM, or primary human monocyte-derived macrophages (HMDM), to investigate the contribution of prolonged stimulation with C1q on macrophage efferocytosis and production of proinflammatory cytokines. Mouse and human macrophages were adhered to C1q for either 30 min or 18 h (prolonged stimulation), followed by a 4 h stimulation with LPS, and production of TNFα and IL-6 was measured by ELISA. While mouse macrophages required prolonged stimulation with C1q in order to downregulate TNFα production, human macrophages responded immediately to C1q (within 30 min) to dampen LPS-induced TNFα production. Interestingly, IL-6 production was significantly enhanced in HMDM, but only after 18 h programming with C1q (Figure 1). Fraser and colleagues previously reported enhanced IL-6 expression in human monocytes after 18 h stimulation with C1q in the presence of LPS (18). These data suggest that there are multiple mechanisms leading to C1q-dependent regulation of proinflammatory cytokine production involving both immediate responses and responses that require prolonged stimulation. Interestingly, the same is true for C1q-dependent phagocytosis where enhanced phagocytosis of antibody-opsonized particles occurs within minutes of stimulation with C1q (18), whereas C1q also regulates gene expression in macrophages leading to enhanced engulfment of apoptotic cells (5).

Our previous studies demonstrated that prolonged stimulation with C1q resulted in the development of a pro-efferocytic phenotype in mouse BMDM (5). Similar to the mouse cells, human macrophages (but not human monocytes) responded to prolonged stimulation with C1q with enhanced efferocytic function (Figure 2). C1q-dependent enhanced efferocytosis required a change in protein expression since cycloheximide inhibited the pro-efferocytic phenotype. Our previous studies indicated that programmed efferocytosis in mouse BMDM resulted from C1q-dependent expression of Mer tyrosine kinase and Gas6; however, the human macrophages did not upregulate Mer or the related TAM family receptor, Axl, in response to C1q following 18 h stimulation (Figure 4C). Therefore, the mechanism leading to C1q-dependent programmed efferocytosis in mouse is likely different from human; however, different cell culture conditions or time points may reveal a role for TAM receptors that was not detected here. In any case, our data suggest that C1q stimulates the expression of pro-efferocytic molecules in both systems. Consistent with this observation, Majai et al. (10) demonstrated that macrophages from lupus patients have decreased expression of pro-efferocytic molecules. Furthermore, while C1q-dependent engulfment of apoptotic cells required Mer tyrosine kinase, we observed that multiple engulfment molecules were upregulated by C1q in our initial array, including C1q itself and MFG-E8 (5). Therefore, C1q may stimulate expression of different engulfment molecules under different conditions or microenvironments leading to development of a pro-efferocytic phenotype. Since C1q deficiency results in a lupus-like disease [reviewed in Leffler et al. (23)], it is possible that the lack of C1q in lupus patients results in the maturation of macrophages that are defective in clearance of apoptotic cells because they fail to express or upregulate the critical engulfment proteins.

The TAM family of receptors regulate the clearance of apoptotic cells, and this family also regulates the inhibition of proinflammatory signaling (11), similar to C1q. Therefore, we assessed expression of Mer and the related family member, Axl, in BMDM and HMDM. Unlike mouse macrophages, the human macrophages failed to upregulate expression of Mer and Axl indicating that the alteration of cytokine signaling in these cells was independent of Mer/Axl expression. Similarly, expression of LPS receptors TLR4 and CD14 was not altered in C1q-stimulated mouse macrophages (data not shown) indicating that the defect in signaling was downstream of the LPS receptors. Fraser et al. (21) previously demonstrated that C1q stimulated the activity of inhibitory NFκB complexes leading to a dampening of proinflammatory cytokine production in human monocytes, so it is possible that the defect in proinflammatory signaling in human and mouse macrophages occurs at the level of transcription. Our current studies are focusing on the molecular mechanisms of C1q-dependent signaling.

The initial step in the signal transduction pathway downstream of C1q is the binding of C1q to the cell; however, the C1q receptors responsible for mediating C1q-dependent efferocytosis or inflammatory signaling have yet to be identified. We previously demonstrated that C1q-dependent programming of efferocytosis in mouse BMDM was independent of several of the described C1q receptors (5, 15). Therefore, to begin to define the signal transduction pathway required for C1q-dependent programming, we have taken two directions: (1) to identify intracellular signaling pathways downstream of C1q binding (15) and (2) to characterize the important domains on C1q that are required to mediate C1q-dependent signaling. Our previous studies demonstrated that C1q-dependent upregulation of efferocytosis requires activation of AMP-activated protein kinase (AMPK) (15), and our preliminary studies suggest that AMPK is similarly activated in HMDM (data not shown). In addition, the collagen-like tails were not sufficient for upregulating expression of Mer or enhancing efferocytosis in BMDM (15). Here, we demonstrated that the full-length molecule was also required for the dampening of TNFα production in both mouse and human macrophages and enhanced efferocytosis in human macrophages, since the collagen-like tails failed to inhibit C1q-dependent downregulation of proinflammatory cytokine production or increase efferocytosis in HMDM (Figure 5). It has not been determined if the C1q globular heads are sufficient for upregulating efferocytosis or regulating cytokine production; however, we demonstrated that adiponectin, a C1q homolog, could also upregulate Mer-dependent efferocytosis (15) and the adiponectin heads were not sufficient for mediating this activity (data not shown). Therefore, these C1q-dependent functions may require a full-length molecule.

It has been known for nearly 30 years that C1q provides an immediate signal for enhancement of phagocytic function (24), and C1q has been studied extensively as a physical bridging molecule between macrophages and apoptotic cells [reviewed in Galvan et al. (2)]. However, more recently, we have begun to appreciate the programming activity of C1q, which suggests that C1q programs macrophages for a pro-resolving and anti-inflammatory phenotype [reviewed in Bohlson et al. (1)]. It is becoming clear that C1q functions through multiple pathways, including the activation of the classical complement pathway, directly opsonizing for phagocytosis, and directly regulating gene expression. Moreover, C1q has different activities in mice and humans since C1q deficiency in humans results in lupus with nearly 100% penetrance, whereas the development of autoimmunity in mice is highly dependent on genetic background (25). Elucidating the molecular pathways involved in C1q-dependent functions in mouse and human cells should help in identifying new methods for targeting autoimmune diseases and chronic inflammation.

## Author Contributions

HH and SO were responsible for experimental design and execution, writing of Materials and Methods, generation of the majority of figures and legends, and critically reviewing the entire manuscript. ES performed the experiments shown in Figures 4A,B, including experimental design and execution, and also critically reviewed the manuscript. CJ performed all of the statistical analysis presented in the manuscript, aided with the design of experiments (advised on replicates), and wrote sections of the manuscript involving Statistical Analysis. SB directed the experiments of the team, analyzed data, wrote the manuscript (aside from sections mentioned above), and edited the manuscript.

## Conflict of Interest Statement

The authors declare that the research was conducted in the absence of any commercial or financial relationships that could be construed as a potential conflict of interest.
