# Supplementary material for: Complement Component C1q Programs a Pro-Efferocytic Phenotype while Limiting TNFα Production in Primary Mouse and Human Macrophages
Source: Front Immunol. 2016 Jun 15;7:230. doi: 10.3389/fimmu.2016.00230 (PMC4908142; doi:10.3389/fimmu.2016.00230)
Supplement: Supplementary file 1 [file Table_1.DOCX]

| Figure number | % live  cells | % apoptotic  cells | % apo-necrotic  cells | |
| --- | --- | --- | --- | --- |
| Fig 2 |  |  |  |  |
| 30min # 1 | 19.9 | 29.3 | 49.2 |  |
| 30 min # 2  30 min #3 | 14.4  9.13 | 56.2  50.2 | 29.0  38.2 |  |
|  |  |  |  |  |
| 18h # 1 | 23.5 | 45.3 | 30.2 |  |
| 18h # 2 | 13.7 | 56.4 | 27.9 |  |
| 18h # 3  18h # 4 | 25.0  19.9 | 48.7  42.7 | 25.9  34.7 |  |
|  |  |  |  |  |
| Fig 3A |  |  |  |  |
| 1 | 16.1 | 83.9 |  |  |
| 2 | 13.8 | 86.2 |  |  |
| 3 | 16.6 | 83.4 |  |  |
| 4 | 15.4 | 84.6 |  |  |
|  |  |  |  |  |
| Fig 5B |  |  |  |  |
| 1 | 16.1 | 46.0 | 35.9 |  |
| 2 | 16.1 | 46.0 | 35.9 |  |

Supplemental table 1. Annexin V and Propidium iodide staining were used to measure percent apoptotic (Annexin V-positive) and apo-necrotic (annexin V and propidium iodide-positive) cells used in experiments shown in figures 2 and 5. Only annexin V staining is was available for figure 3A (% apoptotic cells). Staining for two of three experiments is shown for figure 5B.
