# Supplementary figures and images for: Complement Component C1q Programs a Pro-Efferocytic Phenotype while Limiting TNFα Production in Primary Mouse and Human Macrophages
Source: Front Immunol. 2016 Jun 15;7:230. doi: 10.3389/fimmu.2016.00230 (PMC4908142; doi:10.3389/fimmu.2016.00230)

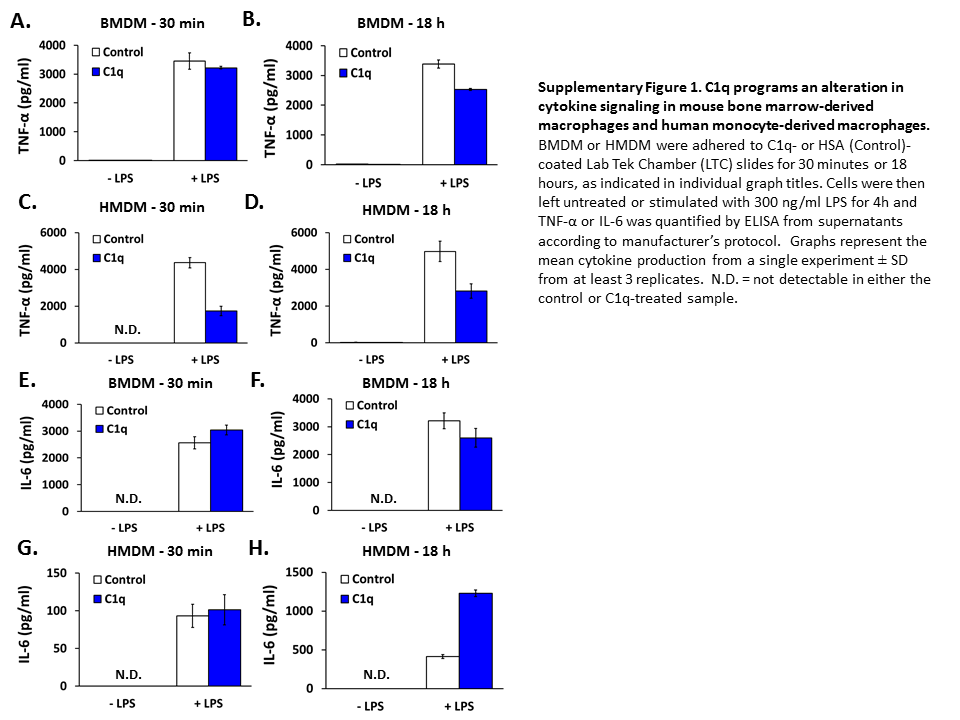

Supplement: Supplementary file 2 [file Image_1.TIF]

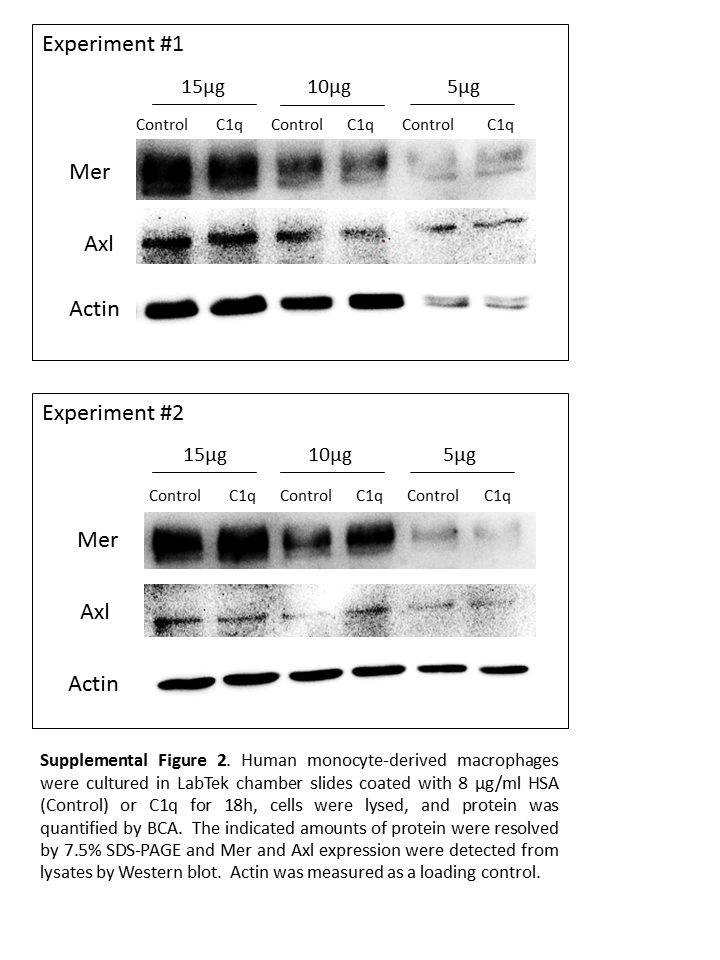

Supplement: Supplementary file 3 [file Image_2.TIF]
